# PLM-eXplain: divide and conquer the protein embedding space

**DOI:** 10.1093/bioinformatics/btaf631

**Published:** 2025-11-21

**Authors:** Jan van Eck, Dea Gogishvili, Wilson Silva, Sanne Abeln

**Affiliations:** AI Technology for Life, Department of Computing and Information Sciences, Department of Biology, Utrecht University, Utrecht, 3584CC, The Netherlands; AI Technology for Life, Department of Computing and Information Sciences, Department of Biology, Utrecht University, Utrecht, 3584CC, The Netherlands; AI Technology for Life, Department of Computing and Information Sciences, Department of Biology, Utrecht University, Utrecht, 3584CC, The Netherlands; AI Technology for Life, Department of Computing and Information Sciences, Department of Biology, Utrecht University, Utrecht, 3584CC, The Netherlands

## Abstract

**Motivation:**

Protein language models (PLMs) have revolutionized computational biology through their ability to generate powerful sequence representations for diverse prediction tasks. However, their black-box nature limits biological interpretation and translation to actionable insights. Bridging this gap requires approaches that maintain predictive performance while providing interpretable explanations of model behaviour.

**Results:**

We present PLM-eXplain (PLM-X), an explainable adapter layer that bridges this gap by factoring PLM embeddings into two complementary components: an interpretable subspace based on established biochemical features, and a residual subspace that retains predictive, non-interpretable information. Using embeddings from ESM2 and ProtBert, PLM-X incorporates well-established properties, including secondary structure and hydropathy, while maintaining high predictive performance. We demonstrate the effectiveness of our approach across three biologically relevant classification tasks: extracellular vesicle association, transmembrane helix prediction, and aggregation propensity prediction. PLM-X enables biological interpretation of model decisions without sacrificing accuracy, offering a generalizable solution for enhancing PLM interpretability across various downstream applications.

**Availability and implementation:**

Source code and models are available at https://github.com/AIT4LIFE-UU/PLM-eXplain/.

## 1 Introduction

The field of computational biology has expanded rapidly, supported by the development of large-scale protein language models (PLMs) trained on extensive sequence databases (Elnaggar *et al.* 2022, Lin *et al.* 2023). These models quickly outperformed existing tools across a variety of protein prediction tasks (Bepler and Berger 2021, Elnaggar *et al.* 2022, Lin *et al.* 2023, Yu *et al.* 2023, Hie *et al.* 2024, Hayes *et al.* 2025), enabling highly accurate predictions of properties ranging from secondary structure (Høie *et al.* 2022, Gogishvili *et al.* 2024) and subcellular location (Thumuluri *et al.* 2022) to protein aggregation (Perez *et al.* 2023). A key innovation behind these PLMs is the transformer architecture (Vaswani *et al.* 2017), which uses multi-head self-attention mechanisms to process entire protein sequences. This allows the model to learn context-dependent representations for each amino acid. This process captures patterns in the sequence that are stored in a numerical representation. The resulting dense embeddings integrate both local and global sequence information, making them valuable for various downstream tasks.

A critical challenge remains that the representations learned by PLMs are not interpretable, in contrast to traditional shallow learning approaches that rely on hand-engineered features (Hou *et al.* 2022). Although traditional methods may be limited in their predictive power, their use of carefully crafted features, such as physicochemical properties and various experimental annotations, provides clear biological meaning to their predictions (Waury *et al.* 2024). PLMs, however, transform protein sequences into high-dimensional amino acid-based representations without specific biological meaning attached, offering limited insight into the underlying principles driving their predictions. This lack of interpretability limits scientific understanding of how PLMs capture biological mechanisms, potentially hindering their integration into experimental workflows where model decisions need clear biological rationales (Vellido 2020, Frasca *et al.* 2024). Efforts to address these issues have included post hoc analyses (Vig *et al.* 2020, Dickinson and Meyer 2022, Hou *et al.* 2023, Pratyush *et al.* 2024), sparse autoencoder approaches (Simon and Zou 2024), and structural mapping strategies (Detlefsen *et al.* 2022). Although valuable, these methods do not provide a direct and reusable partition of the embedding space into biologically meaningful and residual components. In this study, we present a new explainable adapter approach, PLM-eXplain (PLM-X) that retains the prediction power of protein language models while including interpretability of the representations. Our method utilizes embeddings derived from ESM2 (Lin *et al.* 2023) and ProtBert (Elnaggar *et al.* 2022), some of the most advanced PLMs currently available. We factored these embeddings into two complementary components: a subspace composed of crafted, interpretable physicochemical features and a compressed residual subspace capturing information not explicitly described by these known attributes. By anchoring a fraction of the embedding space in known descriptors, such as secondary structure classifications (SS3 and SS8) (Kabsch and Sander 1983) and hydropathy (GRAVY) (Kyte and Doolittle 1982), we empower researchers to better rationalize the contributions of fundamental chemical and structural factors. The remaining subspace ensures that the model retains its full predictive power, preserving more subtle patterns that contribute to performance but are not captured by the predefined feature set. Our explainable adapter is reusable as a flexible layer that can be integrated into various downstream tasks without requiring retraining the adapter. To illustrate this versatility, we applied our semi-explainable embeddings to three distinct protein-level (global) classification problems: (i) prediction of extracellular vesicle (EV) proteins, which are crucial disease biomarkers occurring in all domains of life (Borges *et al.* 2013, Yáñez-Mó *et al.* 2015, van Niel *et al.* 2018, Gámez-Valero *et al.* 2019); (ii) identification of transmembrane proteins, a well-characterised task with state-of-the-art solutions (Hallgren *et al.* 2022); (iii) prediction of protein aggregation propensity (amyloidogenicity), which remains a critical challenge in clinical and biotechnological applications (Chiti and Dobson 2017, Dobson *et al.* 2020, Ke *et al.* 2020). In each of these cases, we demonstrate that our explainable adapter not only preserves the high accuracy characteristic of black-box PLM embeddings, but also provides a better method to explain the model’s decisions.

## 2 Materials and methods

**Figure 1.**
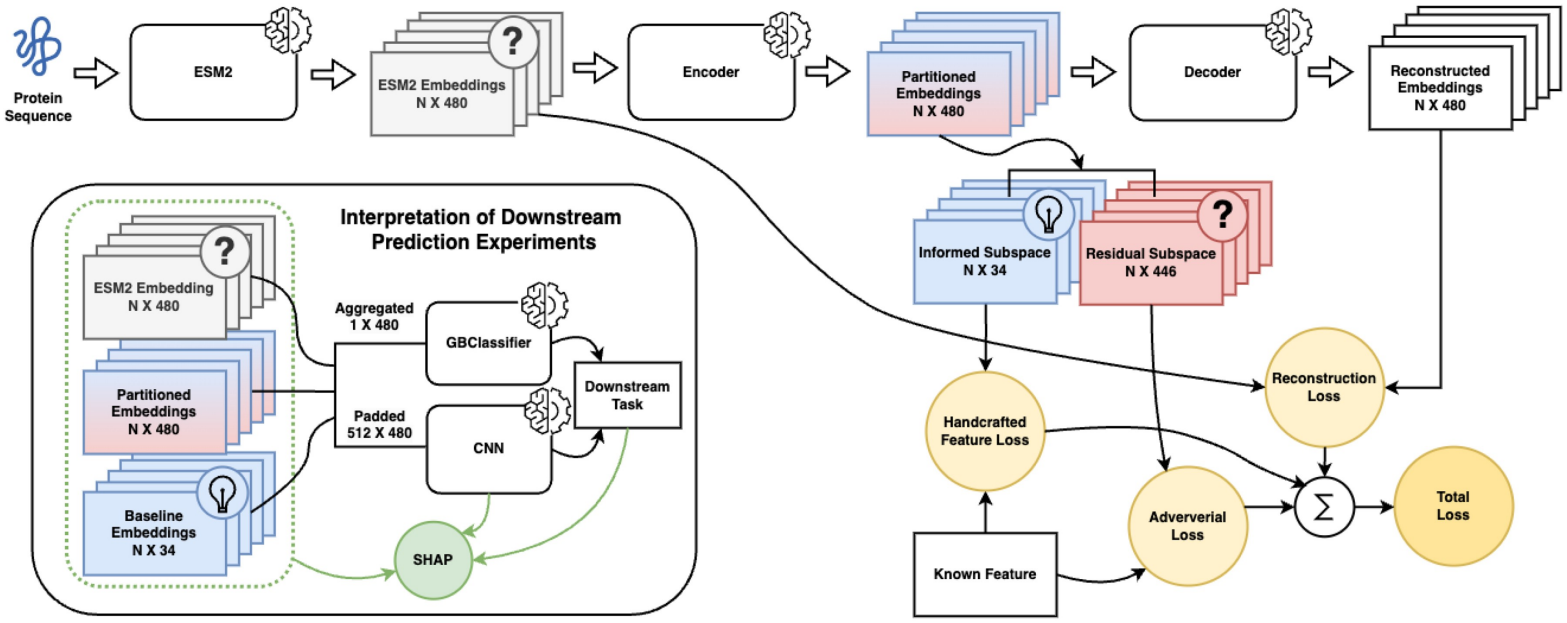
Our encoder-decoder model architecture (ESM2-35M) splits protein language model embeddings into two complementary subspaces: one capturing explicit physicochemical features and another containing the residual predictive information. An adversarial component stimulates this separation while preserving the original embeddings’ prediction capabilities. We evaluate the partitioned embeddings using Gradient Boosting Classifier and CNN models on three downstream protein-level tasks.

Our method uses a two-step approach to enhance the interpretability of protein language models while maintaining their predictive power (Fig. 1). First, we train an adapter layer for the PLM that serves as encoder and transforms traditional PLM embeddings into partitioned representations. This process splits the embedding space into two complementary components: an informed subspace grounded in established biochemical features and a residual subspace that preserves additional predictive information not captured by known attributes. Second, to demonstrate the versatility and effectiveness of these adapted embeddings, we evaluate them across three distinct protein classification tasks: aggregation propensity, EV association, and transmembrane helices classification. For each task, we explore two different architectural approaches: a protein-level (global) analysis method that pools amino acid embeddings by averaging and a local analysis method using convolutional neural networks (CNNs) to capture local patterns of crafted features.

### 2.1 Partitioning PLM embeddings

To transform the ESM2 and ProtBert embeddings (ESM2–35M, ESM2-650M, and ProtBert-bfd) into partitioned representations, we create two complementary subspaces. The combined partitioned embeddings consist of 480, 1280, 1024 features, respectively, matching the size of the original PLM embeddings: an informed subspace that explicitly captures established biochemical features (N X 34), and a residual subspace (N X [original embedding size minus 34 crafted features]) that captures the residual predictive information from the original embedding (Fig. 1).

The informed subspace is designed to represent well-understood protein characteristics in a transparent manner. By incorporating (hand)crafted features based on fundamental biochemical properties, this subspace provides direct interpretability for a portion of the model’s decision-making process. These features include the following: hydrophobicity scales (GRAVY), aromaticity, secondary structure components (SS3, SS8), accessible surface area (ASA) (Miller *et al.* 1987) and standard amino acid types (Table 1, available as supplementary data at *Bioinformatics* online describes crafted features in detail). Each latent feature within this subspace corresponds to a distinct known element.

In contrast, the residual subspace preserves information that cannot be readily explained by biochemical features analysed in this work. This subspace is trained adversarially to promote separation with the knowledge-informed subspace while maintaining the predictive power of the model. This design ensures that subtle patterns and complex relationships in the protein sequence data are not lost in the pursuit of interpretability (Fig. 1).

### 2.2 Model architecture

To maintain the fidelity of the original embeddings, we use an auto-encoder architecture with specific optimization objectives (Fig. 1): (i) the encoder transforms the amino acid embeddings into our partitioned representation, ensuring that handcrafted features are distinctly captured in the informed subspace. (ii) Adversarial training is applied to promote separation between interpretable and non-interpretable components by penalizing the presence of handcrafted features information in the residual subspace. (iii) The decoder reconstructs the original PLM embedding from our partitioned representation, ensuring that no essential information from the original embedding is lost during the transformation process. The resulting architecture (Fig. 1) creates a bridge between the powerful partitioned representations learned by the PLMs and the need for biological interpretability, while preserving the full content of information from the original embeddings. A more detailed overview of the model is shown in Fig. 1, available as supplementary data at *Bioinformatics* online.

To achieve the partitioned representation, a dual branch architecture is applied to the encoder. The informed subspace branch uses two fully connected layers with 480, 1280, or 1024 neurons in the first layer, and 34 neurons in the second layer to map the predefined handcrafted features. The dimension of the original embedding was chosen for the first layer to prevent compression while maintaining efficiency. The first layer is followed by a RELU activation, while the last layer is followed by a Tanh function. Each separate prediction task was trained with its own singular scaling parameter to enable accurate predictions for large numerical ranges. The residual subspace branch consists of two fully connected layers, each having the size of the original embedding in the first layer and the size of the original embedding minus 34 neurons in the second layer, followed by the same activations as the informed subspace branch. The decoder reconstructs the original PLM embeddings from the concatenated partitioned latent representation. The architecture includes two fully connected layers, each with the size of the original embedding, followed by RELU activation. Adversarial training is implemented on the residual subspace using task-specific adversarial networks in combination with Gradient Reversal Layers (GRLs) (Ganin *et al.* 2016). Each adversarial network receives the residual subspace as input and first projects it through a dense layer of half the dimensionality of the original embedding, followed by a ReLU activation. This is then mapped to a 34D dense layer aligned with the handcrafted feature space, ensuring direct coupling to the known feature prediction task. In the forward pass, the GRL transmits the embeddings to the adversarial network without modification. In the backward pass, it scales the encoder gradients by a negative factor.

We evaluated our partitioned embeddings with two complementary approaches to capture both global protein properties and local sequence-level features. The partitioned embeddings were compared against the original ESM2 embeddings and the informed subspace in which the argmax was taken over the multi-label informed subspaces. This approach reflects a realistic baseline where discrete class predictions (such as secondary structure states) are reduced to their most likely class, which is commonly done in practical applications. In addition to the crafted only baseline, we also evaluated the informed subspace directly.

For global verification, we train a Gradient Boosting Classifier with sequence averaged partitioned embeddings on the three different downstream tasks. The Gradient Boosting Classifier was configured with a maximum of 100 iterations, a maximum depth of 4, and a learning rate of 0.05. For the validation of the local context, we implemented a convolutional neural network (CNN) architecture. The CNN consisted of a single convolutional layer with 50 filters, a dropout of 0.2, a learning rate of 0.0001 and a kernel size of 6 for aggregation and 8 for EV and transmembrane helix prediction. This layer is followed by a ReLU activation and a max pooling operation (MaxPool1D). A single feed-forward layer is applied onto the pooled features. The model training process was conducted over a maximum of 20 epochs, where the best-performing model was selected based on the validation set. Training was performed with a batch size of 16. The confidence intervals were calculated by bootstrapping the training data for 10 rounds per experiment to validate the robustness of the embeddings, using 95% $t_{\text{CI}\;\text{of}\;\text{mean}}$.

### 2.3 Loss functions

The development of PLM-X is guided by three distinct loss functions, each serving a specific purpose in the creation of our partitioned embeddings (Fig. 1). Individual loss functions are themselves composites of multiple feature-specific losses, tailored to the nature of each predicted attribute. For multi-class classification tasks, such as secondary structure prediction (SS3 and SS8), we use cross-entropy loss. For binary classification we use binary cross entropy. We used the L1 loss for continuous features, including ASA and GRAVY.

First, we use a handcrafted feature loss ($L_{\text{hcf}}$) that ensures that the informed subspace accurately captures predefined biochemical features. This loss function measures the difference between the predicted and actual values of our crafted features, encouraging the model to learn explicit representations of these established protein characteristics. Second, an adversarial feature loss ($L_{\text{adv}}$) is implemented to maintain the knowledge separation of the two subspaces. Third, a reconstruction loss ($L_{\text{rec}}$) verifies that the combined information from both subspaces reproduces the original PLM embeddings. This loss function measures the discrepancy between the decoder’s output and the initial embeddings, ensuring that no essential information is lost during the transformation process.


(1) $$L_{\text{rec}}=L_{\text{rec}}\left(z_{\text{orig}},z_{\text{recon}}\right),$$

(2) $$L_{\text{adv}}^{\left(t\right)}=L_{\text{adv}}^{\left(t\right)}\left(f_{\text{real}}^{\left(t\right)},f_{\text{pred}}^{\left(t\right)}\right),$$

(3) $$L_{\text{hcf}}^{\left(t\right)}=L_{\text{hcf}}^{\left(t\right)}\left(f_{\text{real}}^{\left(t\right)},f_{\text{pred}}^{\left(t\right)}\right),$$

(4) $$L_{\text{total}}=\lambda_{\text{rec}}L_{\text{rec}}+\sum_{t=1}^{T}\lambda_{\text{hcf}}^{\left(t\right)}L_{\text{hcf}}^{\left(t\right)}+\sum_{t=1}^{T}\lambda_{\text{adv}}^{\left(t\right)}L_{\text{adv}}^{\left(t\right)}.$$

The hyperparameter $\lambda_{\text{rec}}$ represents the weight for the reconstruction loss (1) ($L_{\text{rec}}(z_{\text{orig}},z_{\text{recon}})$), where $z_{\text{orig}}$ is the original embedding produced by the encoder, and $z_{\text{recon}}$ is the reconstructed embedding. The terms $\lambda_{\text{hcf}}^{\left(t\right)}$ and $\lambda_{\text{adv}}^{\left(t\right)}$ denote the weights for the feature loss and feature specific adversarial loss (2) ($L_{\text{hcf}}^{\left(t\right)}$) and adversarial loss (3) ($L_{\text{adv}}^{\left(t\right)}$), respectively, for the *t*th specific task. Here, $f_{\text{real}}^{\left(t\right)}$ represents the real (ground-truth) feature for task *t*, and $f_{\text{pred}}^{\left(t\right)}$ represents the corresponding predicted feature. The term *T* corresponds to the total number of tasks. The total loss (4) ($L_{\text{total}}$) is expressed as a weighted sum of the three components, where the hyperparameters $\lambda_{\text{rec}}$, $\lambda_{\text{hcf}}^{\left(t\right)}$, and $\lambda_{\text{adv}}^{\left(t\right)}$ regulate the contribution of the reconstruction, feature, and adversarial losses, respectively (Fig. 1).

### 2.4 Data collection and curation

We adapted our model using 542 378 Swiss-Prot protein structures from AlphaFoldDB (accessed 18 March 2025), filtered to 25% sequence similarity (Jumper *et al.* 2021, Varadi *et al.* 2024). For each amino acid, we computed structural and physicochemical features using DSSP (Kabsch and Sander 1983), including eight-state (SS8) and three-state (SS3) secondary structures, as well as solvent accessibility (ASA) (Fig. 3, available as supplementary data at *Bioinformatics* online). Additional properties, such as GRAVY scores (Kyte and Doolittle 1982) and aromaticity, were computed with BioPython (Cock *et al.* 2009). We also included one-hot encodings for the 20 standard amino acids (Table 1, available as supplementary data at *Bioinformatics* online). These features were selected both for their fundamental biochemical relevance and because of their established involvement in the downstream tasks evaluated in this study. After filtering, the final dataset comprised 49 543 proteins. ESM2 embeddings were generated for each protein, and residues with pLDDT scores (a per residue measure of local confidence) below 0.7 were excluded from training PLM-X. By excluding residues with pLDDT below 0.7, we ensured high-confidence structural annotations for handcrafted feature labels. However, this choice may bias the dataset towards ordered regions and underrepresent intrinsically disordered regions.

From the final dataset, 5% of the proteins were held out as a test and validation set, and an additional 5% were set aside as a training subset to assess the effectiveness of the adversarial training. This validation was performed by training a two-layer neural network on the residual subspace to predict handcrafted features. The resulting predictive performance served as a proxy for the extent to which handcrafted information had been suppressed from the residual representation.

We selected three biologically significant prediction tasks to evaluate our partitioned embeddings: protein aggregation propensity, EV association, and transmembrane helix prediction. These global (protein-level) binary tasks represent diverse challenges in protein sequence analysis, each requiring the detection of distinct physicochemical and structural features, allowing us to assess the biological relevance of the learned representations. For this, we collected three independent datasets. For the EV association protein prediction, we used a recently curated human proteome dataset (Waury *et al.* 2024). The predictions of protein aggregation propensity were evaluated using the extensively validated amyloid dataset from WALTZ-DB 2.0 (Louros *et al.* 2020). Transmembrane helix proteins were extracted from DeepTMHMM training data. (Hallgren *et al.* 2022).

### 2.5 Model interpretation

The partitioned embeddings were analysed using two complementary methods: SHAP analysis and filter activation-based interpretation. These approaches provided detailed insights into model predictions by quantifying feature importance and exploring the relationship between input sequences and model activations. SHapley Additive exPlanations (SHAP) (Lundberg and Lee 2017) were used to evaluate the contribution of each feature within the partitioned embedding space to model predictions. We used the TreeExplainer module to compute SHAP values for predictions made by the Gradient Boosting classifier on pooled amino acid embeddings. For local interpretation, we analysed the most activated filter in a 1D CNN trained on amino acid-level embeddings for the transmembrane prediction task, focusing on Leptin (AF-P41159-F1). SHAP values were computed over the input sequences and the activations of this filter, providing insights into the sequence regions most relevant to the model’s decisions.

## 3 Results and discussion

The aim of this work is to introduce an explainable adapter to effectively balance PLM interpretability while maintaining predictive power. An initial step of our approach was to train an encoder and transform PLM amino acid embeddings into partitioned embeddings. For this, first, we examined whether the encoder captured handcrafted features distinctly in a respective subspace. In parallel, the residual subspace was adversarially trained to minimize the information about handcrafted features. The decoder reconstructed the original embedding from the partitioned embedding to ensure the conservation of all the information.

**Figure 2.**
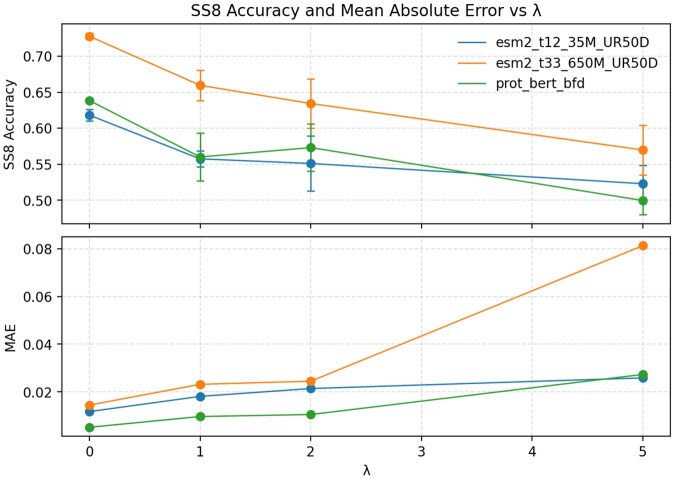
Accuracy and reconstruction MAE for eight classes of secondary structure (SS8). SS8 is predicted from the residual embeddings of PLM-X trained with different adversarial loss weights λ. Top: SS8 accuracy as a function of λ where increasing λ suppresses SS8 signal in the residual space, reducing accuracy. Bottom: reconstruction MAE versus λ where the same increase in λ raises reconstruction error. This illustrates the trade-off between disentanglement (stronger suppression) and reconstruction of the embedding.

To assess adversarial training, we compared the predictive performance of original PLM embeddings and residual subspaces across handcrafted protein features. Figure 2 shows suppression of SS8 in the residual space: higher adversarial weight improved suppression, but increased reconstruction loss. This shows the trade-off between disentanglement and reconstruction. Beyond weight 5, suppression plateaued for all features (Fig. 2, available as supplementary data at *Bioinformatics* online).

At weight 1, reconstruction loss was lowest while handcrafted features were partly suppressed, suggesting downstream tasks rely more on the informed subspace. The mean absolute reconstruction errors (MAE) for ESM2-35M, ESM2-650M, and ProtBert were 0.018, 0.023, and 0.010, respectively. This shows good preservation is kept in the full embedding space.

### 3.1 Model performance

Having established the separation of embeddings, we aimed to assess the versatility of PLM-X and the possibility to integrate it into different prediction problems. For this, we evaluated two distinct architectural approaches: a pooled embeddings model in which protein embeddings are averaged, and a CNN model using sliding windows throughout the sequence (Table 1). These models were tested across three global binary classification tasks: aggregation propensity, EV association, and transmembrane helix prediction. We selected case examples based on both their biological significance and the availability of high-quality curated datasets. As shown in Table 1, the results highlight that partitioned embeddings perform on par with original ESM2-35M embeddings, while receiving a higher performance compared to informed and crafted features only. Tables 3 and 4, available as supplementary data at *Bioinformatics* online show similar trends for the ESM2-650M and ProtBert backbone. The computational overhead of the two-branch adapter is minimal, adding around 2–3 ms per 300-residue protein on a modern GPU, depending on the PLM backbone. This makes the method applicable even in resource-constrained settings.

**Table 1.** Performance comparison between pooled embeddings and CNN across different prediction tasks for the ESM2 35M backbone.^a^

		Pooled embeddings			CNN		
Prediction task	Embeddings	ROC-AUC	Accuracy	F1	ROC-AUC	Accuracy	F1
Aggregation propensity	Partitioned (PLM-X)	0.89±0.00	0.82±0.01	0.74±0.01	0.88±0.00	0.81±0.02	0.76±0.01
	Original (ESM2)	0.88±0.01	0.81±0.01	0.72±0.02	0.88±0.00	0.81±0.01	0.76±0.01
	Crafted only (baseline)	0.88±0.01	0.81±0.01	0.72±0.01	0.87±0.00	0.79±0.01	0.74±0.01
	Informed subspace	0.88±0.01	0.82±0.01	0.74±0.02	0.87±0.00	0.77±0.01	0.73±0.01
EV association	Partitioned (PLM-X)	0.77±0.00	0.73±0.00	0.57±0.01	0.78±0.00	0.69±0.01	0.63±0.02
	Original (ESM2)	0.78±0.00	0.73±0.00	0.58±0.00	0.78±0.00	0.71±0.01	0.63±0.01
	Crafted only (baseline)	0.74±0.00	0.70±0.00	0.50±0.01	0.72±0.00	0.65±0.03	0.57±0.03
	Informed subspace	0.74±0.00	0.72±0.00	0.53±0.01	0.73±0.00	0.64±0.03	0.58±0.03
Transmembrane helix	Partitioned (PLM-X)	0.99±0.00	0.98±0.00	0.92±0.01	1.00±0.00	0.98±0.01	0.94±0.02
	Original (ESM2)	0.98±0.00	0.98±0.00	0.92±0.01	0.99±0.00	0.98±0.00	0.95±0.00
	Crafted only (baseline)	0.97±0.00	0.96±0.00	0.86±0.02	0.98±0.00	0.97±0.00	0.88±0.01
	Informed subspace	0.97±0.00	0.97±0.00	0.89±0.01	1.00±0.00	0.98±0.00	0.92±0.01

a Values are mean ± 95% confidence interval.

### 3.2 Global interpretation

Our case examples were chosen on the basis of the general understanding of the biological problem and the quality of the available curated datasets. Our main objective was to match the performance between the original PLM embedding and the partitioned version while regaining the ability to interpret predictions on downstream tasks (Table 1).

**Figure 3.**
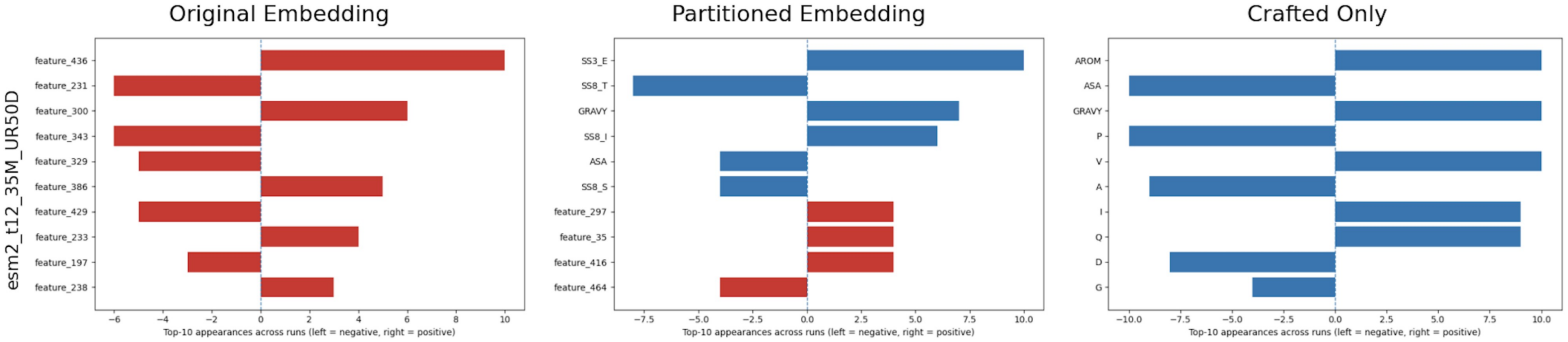
Global feature importance for predicting protein aggregation, comparing three different embedding approaches: the original (ESM2) embeddings, partitioned embeddings (PLM-X), and crafted-only embeddings. For the original model, top features are unknown. For the adapted model, several known features, such as secondary structure components (SS3 E, SS3 T, and SS8 I) and Gravy, can be identified. The bars reflect the sum of the top 10 feature appearances across training bootstrap runs. Negative contributions are shown on the left, positive contributions on the right; blue indicates the informed feature space, and red the residual. Figures 4–6, available as supplementary data at *Bioinformatics* online show detailed top-occurrence SHAP plots for all three downstream prediction tasks.

**Figure 4.**
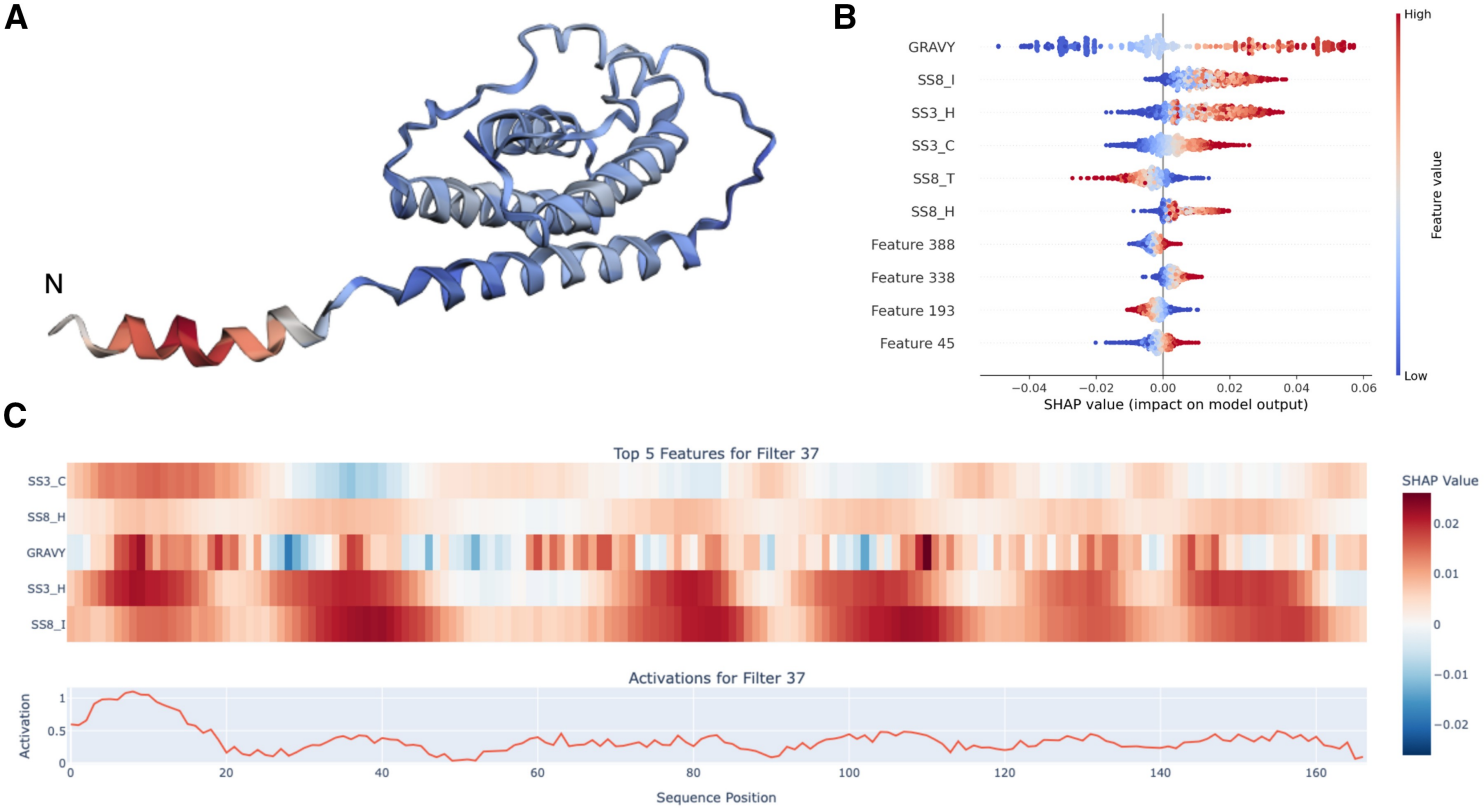
Local interpretation for the transmembrane helix predictions for the Leptin protein. (A) The full-length structure of leptin (AF-P41159-F1), highlighted regions are colour-coded based on the highest activation by Kernel 37. The N terminus (residue 0) is indicated with a small “N.” (B) The most informative features determined by the sum of absolute SHAP values. Each dot represents a feature at a specific position within a motif. (C) Summed SHAP values for a filter, showcasing the top 5 features at each position. This plot highlights the positional importance of features along the activation values.

For global interpretability, the SHAP plots reveal key features driving the predictions for each of our case examples (Figs 4–6, available as supplementary data at *Bioinformatics* online). Although the top features of the original embeddings remain abstract and uninterpretable, our partitioned embeddings identified several biologically relevant properties as important predictors. For the aggregation propensity prediction, GRAVY index emerged as a crucial feature along with the secondary structure component β-strands (SS3 E) across different backbone models, agreeing with the known link between amyloidogenicity and hydrophobicity (Van Gils *et al.* 2020, Thompson *et al.* 2024) (Fig. 3).

For transmembrane helix prediction, well-established features of transmembrane helices, such as the GRAVY index and SS3 helical structures, show clear positive shifts for the ESM2 models. the alpha-helical π-helix (SS8_I) emerged as third most influential known features for the ProtBert based model. Transmembrane regions are typically hydrophobic and adopt stable alpha-helical conformations within the lipid bilayer. It has been shown that π-helices are distinctive for transmembrane proteins (Ludwiczak *et al.* 2019), and is supported in the predicted π-helix distribution of the transmembrane helical dataset (Fig. 7, available as supplementary data at *Bioinformatics* online). For predicting protein sorting in EVs, Cysteine (C) (ESM2 35M) and Histidine (H) (ESM2 35M, ProtBert) was among the top features (Fig. 5, available as supplementary data at *Bioinformatics* online). These findings are consistent with prior research (Waury *et al.* 2024). We note that EV relevant post-translational modifications, which are known strong associators (Waury *et al.* 2024), are not included in our informed subspace. Their signal is therefore likely to be captured by the residual space rather than exposed as interpretable features.

Hence, from the partitioned embeddings (PLM-X), we can learn to what extent the high performing model is based on currently understood biophysical properties. In the case of aggregation prediction, we can conclude that the signal in the training dataset is dominated by beta-strand propensity and hydrophobicity, as well as the residual embedding (Feature 436 in Fig. 3).

### 3.3 Local interpretation

The CNN architecture enables detailed analysis of amino acid-level features and sequence motifs, providing deeper insights into how specific sequence patterns and local structural elements influence the model’s predictions. The transmembrane prediction of Leptin was analysed to understand the contributions of specific features to the predictions made by our model (Fig. 4A–C). For the most activated filter (filter 37), SHAP values were calculated on the partitioned embeddings, providing insights into feature importances (Fig. 4B). This analysis revealed that the GRAVY index and alpha-helix features were the most informative predictors across the sequence. While this example highlights the most activated kernel, interpretations can vary across filters. Each may capture different or complementary biological patterns. Moreover, SHAP scores in regions with low activation should be interpreted with caution. The importance of features can shift across the sequence depending on the strength of the activation.

### 3.4 Limitations

Although PLM-X provides interpretable insights in multiple prediction tasks, several limitations should be acknowledged. First, PLM-X relies on predefined handcrafted features of known potential explanations, on the other hand, the residual subspace remains opaque as its features do not directly map to known biophysical concepts.

Second, the disentanglement enforced by the adversarial objective introduces a trade-off with reconstruction accuracy, raising the possibility that some biologically relevant information is distorted, although this was not observed on the downstream tasks. In addition, although adversarial suppression reduces overlap, traces of informed features remain in the residual subspace. This still allows them to be used instead of the intended informed representation.

Finally, differences between backbone models suggest that interpretability is model-dependent, and explanations may not generalize uniformly across architectures or sequence contexts. Future work should expand the feature set, explore more diverse prediction tasks, and refine the disentanglement objective to better balance interpretability with predictive performance.

## 4 Conclusion and future outlook

In this study, we present an innovative approach for interpreting protein language models. We used three existing different PLM embeddings (ESM2-35M, ESM2-650M, ProtBert) and factored them into two complementary components: a subspace composed of hand-crafted, interpretable features and a compressed residual subspace capturing information not explicitly described by these known attributes. To demonstrate our use-cases, we applied our partitioned embeddings to three distinct classification problems and explained model predictions both on amino acid and protein levels. We showed that our explainable adapter predicts with high accuracy and most importantly provides a possibility to explain the decision of the model. Our explainable adapter provides a versatile foundation that can be applied to a wide range of downstream prediction tasks. PLM-X can be used without requiring any retraining of the original PLM or the adapter. The embeddings generated by the PLM-X adapter can be applied directly to any downstream task, regardless of the type of machine learning model architecture.

This study addresses a fundamental challenge in computational biology, the trade-off between model performance and interpretability. By maintaining high prediction accuracy while providing meaningful biological insights, PLM-X offers a promising direction for developing more trustworthy and actionable AI tools in biological research. Nevertheless, our approach has limitations: it relies on predefined handcrafted features, and the residual subspace remains opaque as its features do not directly map to known biophysical concepts.

Future work could explore the integration of additional physicochemical features and structural information, such as torsion angles or protein surface characteristics. For scenarios where residual latent features emerge as significant predictors, systematic correlation analysis with known biological properties could reveal new insights. The following approaches, such as sparse auto encoders (Simon and Zou 2024), could help identify whether these features represent novel biological concepts or combinations of known properties in superposition. This is particularly valuable for expanding our understanding of how PLMs encode biological information and potentially discovering new protein motifs or structural patterns.

## Supplementary Material

btaf631_Supplementary_Data

## Data Availability

Data and code implemented in this study are available at: https://github.com/AIT4LIFE-UU/PLM-eXplain.
