# Supplementary material for: PLM-eXplain: divide and conquer the protein embedding space
Source: Bioinformatics. 2025 Nov 21;42(1):btaf631. doi: 10.1093/bioinformatics/btaf631 (PMC12790820; doi:10.1093/bioinformatics/btaf631)
Supplement: btaf631_Supplementary_Data [file btaf631_supplementary_data.pdf]

Supplementary Information

Supplementary tables

Crafted Features	Description
ASA	Accessible Surface Area.
SS8_H	Alpha-helix in 8-class secondary structure prediction.
SS8_E	Beta-strand in 8-class secondary structure prediction.
SS8_G	3-10 helix in 8-class secondary structure prediction.
SS8_I	Pi-helix in 8-class secondary structure prediction.
SS8_B	Beta-bridge in 8-class secondary structure prediction.
SS8_T	Turn in 8-class secondary structure prediction.
SS8_S	Bend in 8-class secondary structure prediction.
SS8_-	Coil in 8-class secondary structure prediction.
SS3_H	Alpha-helix in 3-class secondary structure prediction.
SS3_E	Beta-strand in 3-class secondary structure prediction.
SS3_C	Coil in 3-class secondary structure prediction.
A	Amino acid: Alanine.
C	Amino acid: Cysteine.
D	Amino acid: Aspartic Acid.
E	Amino acid: Glutamic Acid.
F	Amino acid: Phenylalanine.
G	Amino acid: Glycine.
H	Amino acid: Histidine.
I	Amino acid: Isoleucine.
K	Amino acid: Lysine.
L	Amino acid: Leucine.
M	Amino acid: Methionine.
N	Amino acid: Asparagine.
P	Amino acid: Proline.
Q	Amino acid: Glutamine.
R	Amino acid: Arginine.
S	Amino acid: Serine.
T	Amino acid: Threonine.
V	Amino acid: Valine.
W	Amino acid: Tryptophan.
Y	Amino acid: Tyrosine.
GRAVY	Grand Average of Hydropathy (measure of hydrophobicity).
AROM	Aromaticity (True for Phenylalanine, Tyrosine and Tryptophan, false for other amino acids).

Table S1. Crafted feature codes and their descriptions.

Metric	ESM2-35M	ESM2-650M	ProtBert
F1 Amino Acid	1.00	1.00	1.00
F1 SS3	0.83	0.89	0.84
F1 SS8	0.67	0.78	0.71
F1 Aromaticity	1.00	1.00	1.00
R^2 ASA	0.45	0.63	0.53
R^2 GRAVY	1.00	1.00	1.00
MAE Reconstruction	0.02	0.02	0.01

Table S2. Performance metrics across three embedding models: classification (F1 scores), regression (R^2), and reconstruction (MAE).

Table S3. Performance comparison between pooled embeddings and CNN across different prediction tasks for PLM-X-ESM2-650M. Values are mean and ± 95% CI.

Prediction task	Embeddings	Pooled embeddings			CNN		
		ROC-AUC	Accuracy	F1	ROC-AUC	Accuracy	F1
Aggregation propensity	Partitioned (PLM-X)	0.88 ± 0.00	0.82 ± 0.01	0.74 ± 0.02	0.88 ± 0.00	0.81 ± 0.02	0.76 ± 0.02
	Original (ESM2)	0.89 ± 0.01	0.83 ± 0.01	0.75 ± 0.01	0.89 ± 0.00	0.82 ± 0.01	0.77 ± 0.00
	Crafted Only (Baseline)	0.88 ± 0.01	0.80 ± 0.01	0.71 ± 0.01	0.88 ± 0.00	0.79 ± 0.01	0.74 ± 0.00
	Informed Subspace	0.88 ± 0.00	0.82 ± 0.01	0.74 ± 0.01	0.87 ± 0.00	0.80 ± 0.01	0.75 ± 0.01
EV association	Partitioned (PLM-X)	0.79 ± 0.00	0.74 ± 0.00	0.60 ± 0.00	0.80 ± 0.00	0.70 ± 0.02	0.65 ± 0.01
	Original (ESM2)	0.80 ± 0.00	0.75 ± 0.00	0.61 ± 0.00	0.79 ± 0.00	0.72 ± 0.01	0.64 ± 0.01
	Crafted Only (Baseline)	0.74 ± 0.00	0.71 ± 0.00	0.51 ± 0.01	0.72 ± 0.00	0.66 ± 0.02	0.59 ± 0.01
	Informed Subspace	0.74 ± 0.00	0.70 ± 0.00	0.49 ± 0.00	0.72 ± 0.00	0.65 ± 0.02	0.58 ± 0.02
Transmembrane helix	Partitioned (PLM-X)	0.99 ± 0.00	0.97 ± 0.00	0.89 ± 0.01	1.00 ± 0.00	0.98 ± 0.01	0.92 ± 0.02
	Original (ESM2)	0.99 ± 0.00	0.97 ± 0.00	0.89 ± 0.01	0.99 ± 0.00	0.99 ± 0.01	0.96 ± 0.02
	Crafted Only (Baseline)	0.97 ± 0.00	0.96 ± 0.00	0.85 ± 0.01	0.98 ± 0.00	0.96 ± 0.00	0.86 ± 0.01
	Informed Subspace	0.97 ± 0.00	0.96 ± 0.00	0.86 ± 0.01	0.99 ± 0.00	0.97 ± 0.01	0.90 ± 0.02

Table S4. Performance comparison between pooled embeddings and CNN across different prediction tasks for PLM-X-ProtBert. Values are mean and ± 95% CI.

Prediction task	Embeddings	Pooled embeddings			CNN		
		ROC-AUC	Accuracy	F1	ROC-AUC	Accuracy	F1
Aggregation propensity	Partitioned (PLM-X)	0.85 ± 0.01	0.78 ± 0.01	0.69 ± 0.02	0.87 ± 0.00	0.81 ± 0.01	0.76 ± 0.01
	Original (ESM2)	0.85 ± 0.01	0.79 ± 0.01	0.69 ± 0.02	0.87 ± 0.00	0.81 ± 0.01	0.75 ± 0.01
	Crafted Only (Baseline)	0.86 ± 0.01	0.79 ± 0.01	0.70 ± 0.02	0.85 ± 0.00	0.78 ± 0.01	0.72 ± 0.01
	Informed Subspace	0.85 ± 0.01	0.79 ± 0.01	0.69 ± 0.02	0.86 ± 0.00	0.78 ± 0.00	0.74 ± 0.00
EV association	Partitioned (PLM-X)	0.78 ± 0.00	0.74 ± 0.00	0.57 ± 0.01	0.79 ± 0.00	0.72 ± 0.01	0.63 ± 0.02
	Original (ESM2)	0.78 ± 0.00	0.74 ± 0.00	0.58 ± 0.01	0.80 ± 0.00	0.73 ± 0.02	0.63 ± 0.01
	Crafted Only (Baseline)	0.73 ± 0.00	0.70 ± 0.00	0.50 ± 0.01	0.72 ± 0.00	0.65 ± 0.03	0.58 ± 0.01
	Informed Subspace	0.75 ± 0.00	0.72 ± 0.00	0.52 ± 0.01	0.74 ± 0.00	0.67 ± 0.02	0.59 ± 0.01
Transmembrane helix	Partitioned (PLM-X)	0.99 ± 0.00	0.98 ± 0.00	0.92 ± 0.01	1.00 ± 0.00	0.99 ± 0.00	0.95 ± 0.01
	Original (ESM2)	0.98 ± 0.00	0.97 ± 0.00	0.88 ± 0.01	1.00 ± 0.00	0.98 ± 0.00	0.95 ± 0.01
	Crafted Only (Baseline)	0.97 ± 0.00	0.96 ± 0.00	0.86 ± 0.01	0.98 ± 0.00	0.96 ± 0.00	0.86 ± 0.01
	Informed Subspace	0.97 ± 0.00	0.97 ± 0.00	0.89 ± 0.01	0.99 ± 0.00	0.98 ± 0.00	0.92 ± 0.01

Supplementary figures

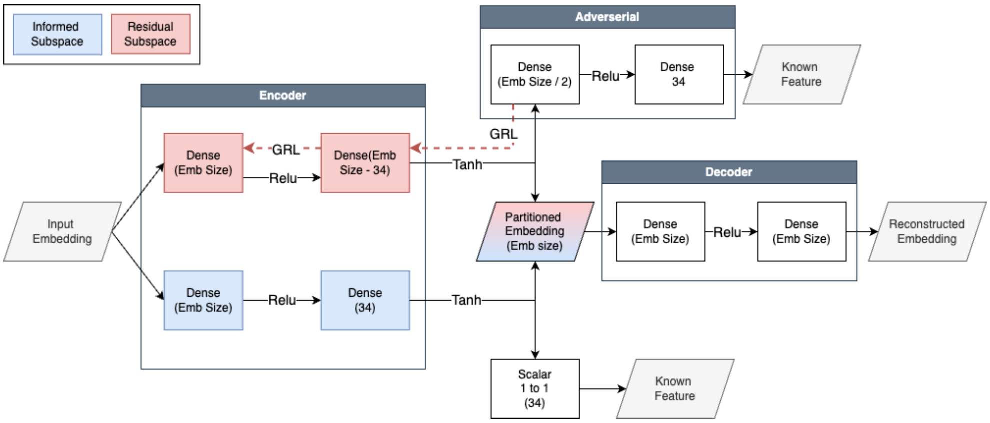

Figure S1. Schematic overview of the PLM-X architecture. The input embedding is encoded into two latent subspaces: an informed subspace and a residual subspace. Trainable scalars are applied to defined index ranges within the known subspace to emphasise biologically meaningful factors. The concatenated latent vector is decoded to reconstruct the original embedding. An adversarial discriminator, connected via a gradient reversal layer, is trained to predict class labels from the unknown subspace, encouraging disentanglement between interpretable and residual features.

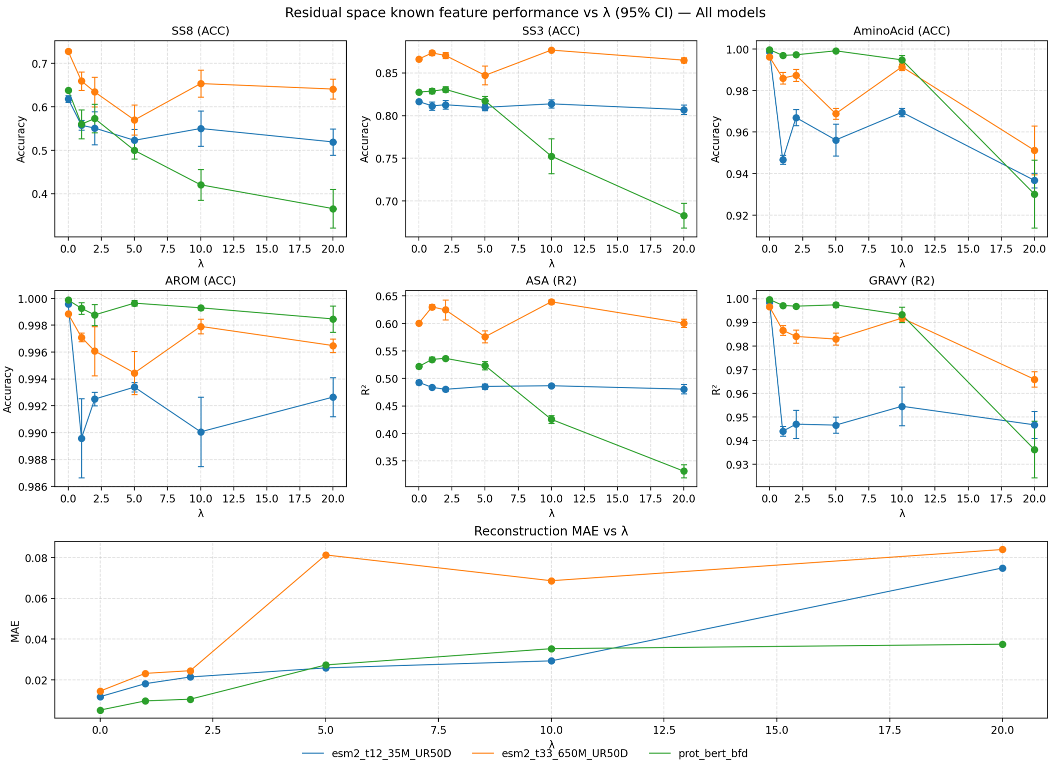

Figure S2. Reconstruction error and residual known feature performance vs adverserial weight The top panels show how increasing the adversarial loss weight (λ) impacts the retention of handcrafted feature information in the residual embedding space. For each λ value, we extracted residual embeddings from PLM-X models and evaluated how well key structural and physicochemical features could be predicted from them. A two-layer MLP was trained separately on each residual embedding variant. The results show a decline in predictive performance as λ increases, indicating that higher adversarial pressure effectively removes handcrafted feature information from the residual space. The bottom panel shows the trade-off with reconstruction performance (MAE). While stronger adversarial pressure promotes disentanglement, it comes at the cost of higher reconstruction error.

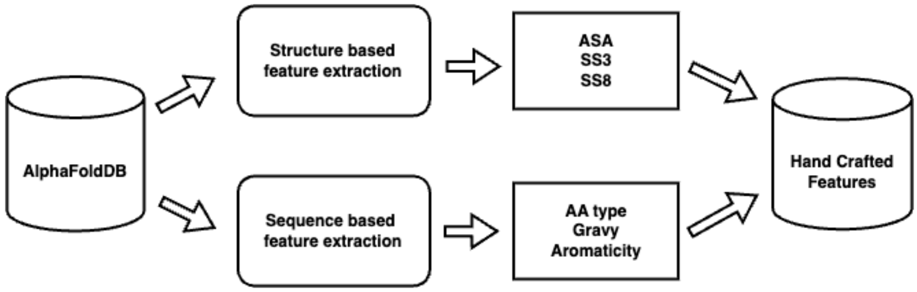

Figure S3. Data curation pipeline for the model adaptation. Human proteome from AlphaFoldDB Jumper et al. 2021; Varadi et al. 2024 was annotated with secondary structure components and other sequence-based features. Resulting 34 features were used to create knowledge informed subspace.

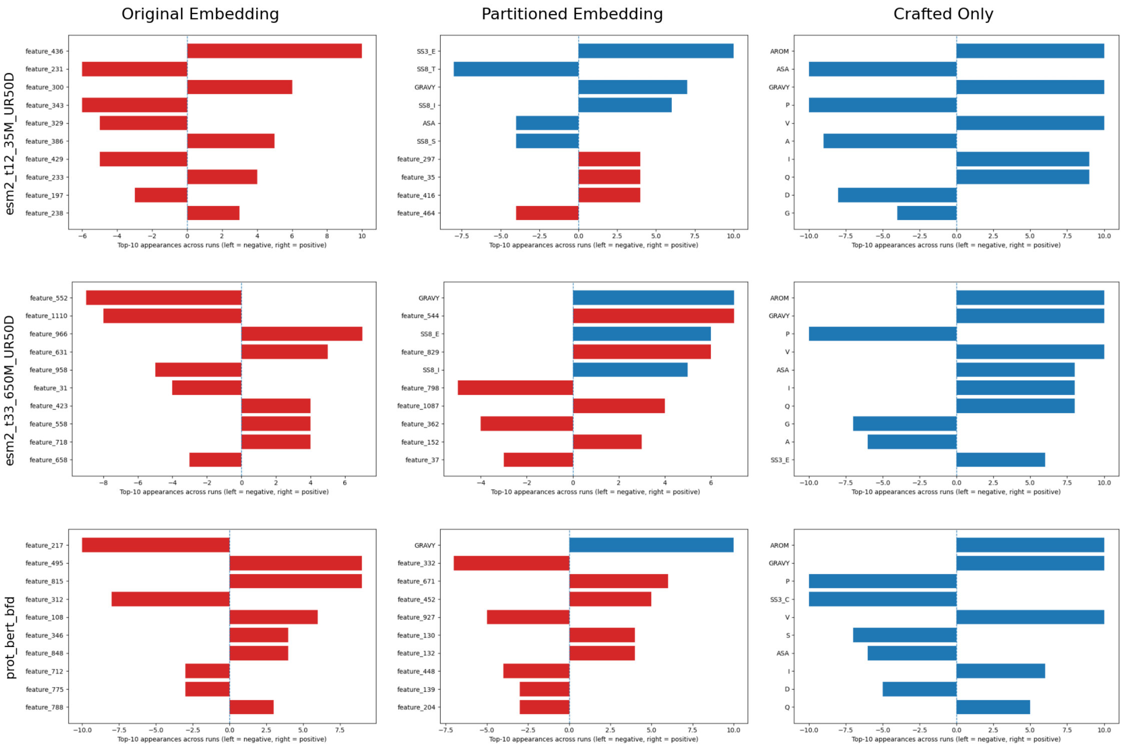

Figure S4. SHAP summary plots for global interpretability of the aggregation propensity prediction task across different backbone models. Columns show results for the original embeddings (left), partitioned embeddings (middle), and crafted-only embeddings (right). Rows correspond to ESM2-35M, ESM2-650M, and ProtBert. Across all backbones, the partitioned embeddings consistently highlight GRAVY as a dominant predictor, in line with its established role in aggregation. In addition, SS3_E emerges as an important feature in both ESM2 backbones, further aligning with known aggregation mechanisms. ProtBert, however, shows weaker recovery of SS3 features and overall lower performance in the pooled embedding setting, which may stem from limitations in capturing SS3_H signals on short hexapeptide sequences.

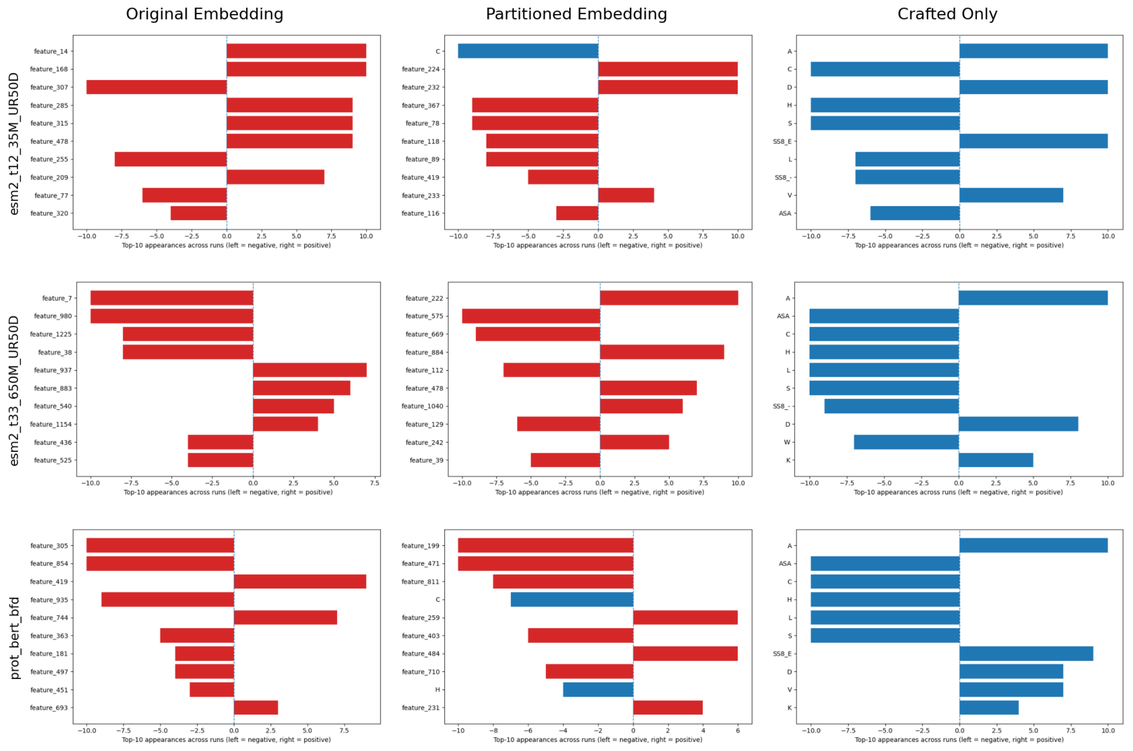

Figure S5. SHAP summary plots for global interpretability of the extracellular vesicle association prediction task across different backbone models. Columns show results for the original embeddings (left), partitioned embeddings (middle), and crafted-only embeddings (right). Rows correspond to ESM2-35M, ESM2-650M, and ProtBert. In the partitioned embeddings, Cysteine (C) consistently emerges as an important feature for both the 35M and ProtBert backbones, whereas the 650M backbone highlights primarily unknown latent features, suggesting that it captures the EV signal in a different way. This indicates that while PLM-X can reliably recover biologically meaningful handcrafted features in some backbones, larger models may rely more heavily on residual, non-interpretable signals which aligns with the performance increase of these larger models.

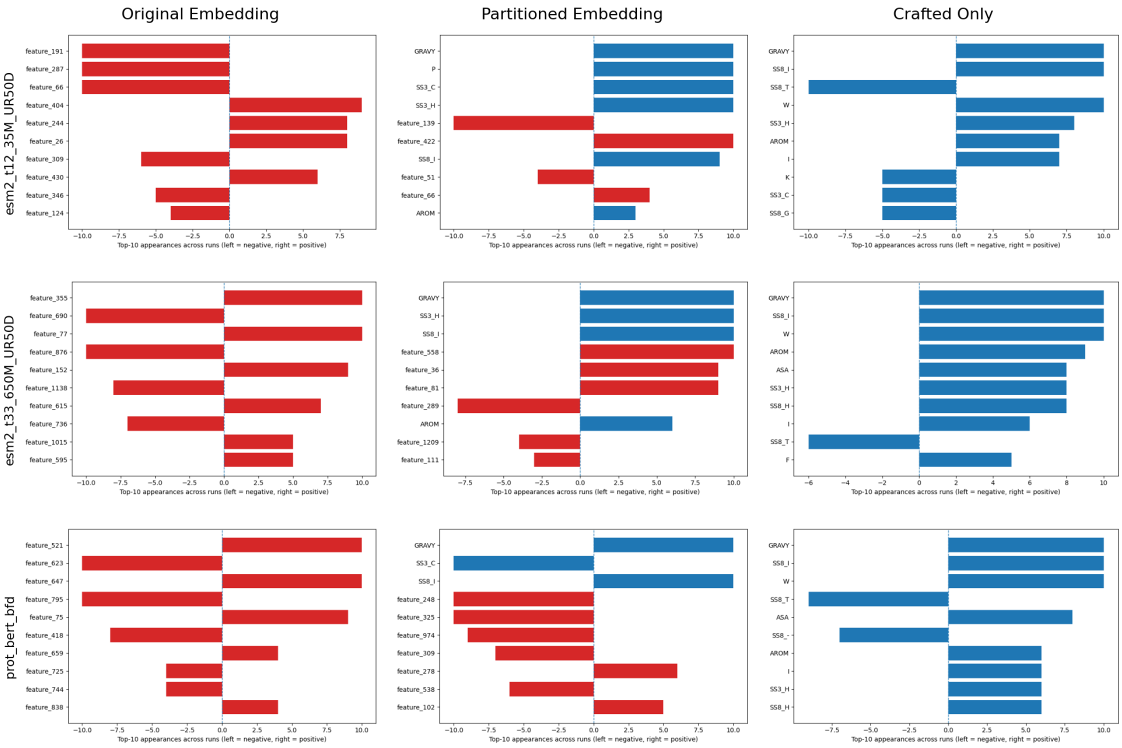

Figure S6. SHAP summary plots for global interpretability of the transmembrane helix prediction task across different backbone models. Columns show results for the original embeddings (left), partitioned embeddings (middle), and crafted-only embeddings (right). Rows correspond to ESM2-35M, ESM2-650M, and ProtBert. Across all three backbones, the partitioned embeddings consistently highlight SS8_I, GRAVY, and SS3_C as important features. Interestingly, ProtBert assigns SS3_C a negative contribution, whereas both ESM2 backbones assign it a positive one, suggesting that this coil-related feature is context dependent and can influence predictions in different directions. In addition, both ESM2 backbones consistently identify SS8_H and SS3_H as relevant, aligning with the known importance of helical features in transmembrane regions. This overlap underscores the robustness of PLM-X in recovering core handcrafted features.

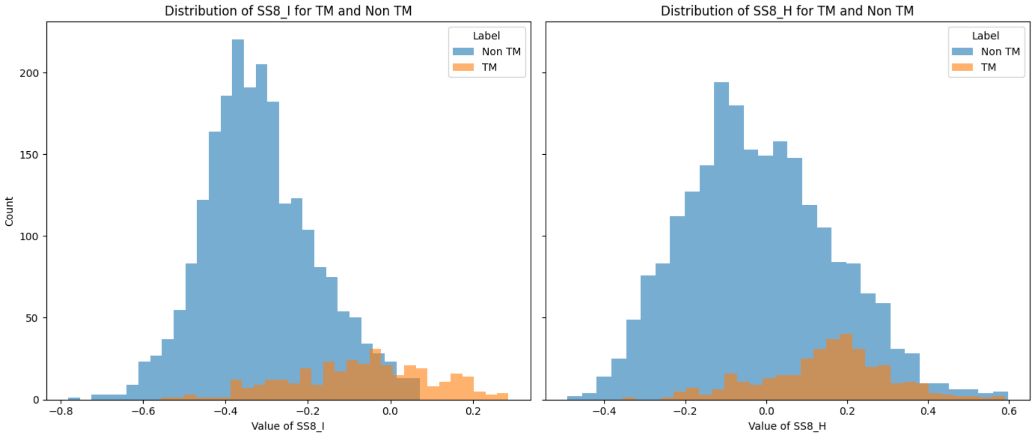

Figure S7. Distribution of predicted concept activations for SS8_I (left) and SS8_H (right) in transmembrane (TM) and non-transmembrane (Non-TM) regions. The Gradient Boosting classifier appears to leverage the absence of SS8_I (left) as a distinguishing signal for non-transmembrane segments, with non-TM regions showing a clear left-skewed distribution centered around -0.4. In contrast, SS8_H (right) reflects a more biologically grounded signal, with transmembrane segments showing a modest right-shift around 0.2. This suggests the model may rely more on easily separable negative signals (low SS8_I) rather than positive indicators (high SS8_H), highlighting a possible representation bias in the learned concept space.
